# Chaperonins: Nanocarriers with Biotechnological Applications

**DOI:** 10.3390/nano11020503

**Published:** 2021-02-17

**Authors:** Sergio Pipaón, Marcos Gragera, M. Teresa Bueno-Carrasco, Juan García-Bernalt Diego, Miguel Cantero, Jorge Cuéllar, María Rosario Fernández-Fernández, José María Valpuesta

**Affiliations:** 1 Centro Nacional de Biotecnología (CNB-CSIC). Darwin, 3. 28049 Madrid, Spain; spipaon@cnb.csic.es (S.P.); mgragera@cnb.csic.es (M.G.); mtbueno@cnb.csic.es (M.T.B.-C.); juanbernalt95@usal.es (J.G.-B.D.); miguel.cantero@uam.es (M.C.); jcuellar@cnb.csic.es (J.C.); mr.fernandez@csic.es (M.R.F.-F.); 2 Unidad Asociada de Nanobiotecnología (IMDEA Nanociencia), 28049 Madrid, Spain

**Keywords:** molecular chaperones, chaperonins, nanocarriers, nanoreactors

## Abstract

Chaperonins are molecular chaperones found in all kingdoms of life, and as such they assist in the folding of other proteins. Structurally, chaperonins are cylinders composed of two back-to-back rings, each of which is an oligomer of ~60-kDa proteins. Chaperonins are found in two main conformations, one in which the cavity is open and ready to recognise and trap unfolded client proteins, and a “closed” form in which folding takes place. The conspicuous properties of this structure (a cylinder containing a cavity that allows confinement) and the potential to control its closure and aperture have inspired a number of nanotechnological applications that will be described in this review.

## 1. Chaperonins: Structure and Function

In the crowded environment of the cell, most proteins need assistance to acquire their native conformation and therefore their functional activity [1]. This task is performed by a group of proteins termed molecular chaperones that are involved in protein homeostasis, which includes assistance in folding, degradation, and prevention of aggregation [2,3]. Molecular chaperones are a large family of proteins, usually heat shock proteins (Hsps), most of which are classified according to the molecular mass of their monomers, though in some cases, they act as oligomers. The most important families are Hsp60, Hsp70, and Hsp90, and even though they are not structurally similar, they each have a dedicated surface for interaction with the client protein and two main conformations: an “open” one that recognizes the protein; and a “closed” one in which it is trapped and somehow helped to find its correct folding. These chaperonins can thus be considered to be molecular machines, and as such their properties could be transformed for nanotechnological purposes [4,5]. This review will focus on the structure and function of one of these families of chaperones, the Hsp60 or chaperonins, and will describe the efforts undertaken to modify their structures for use as nanotechnological devices such as nanocontainers and nanocarriers.

**Figure 1:**
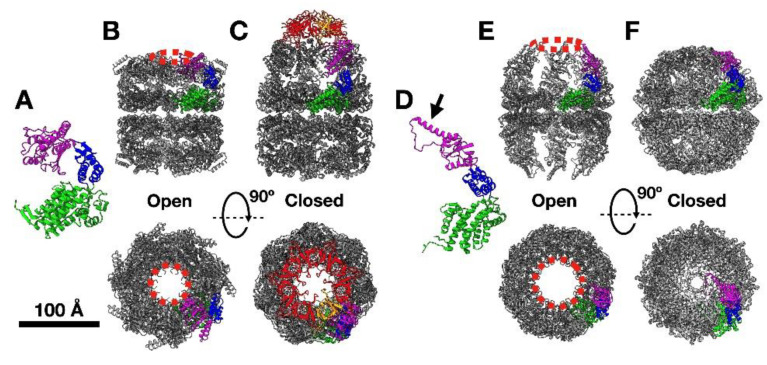
Structure of the chaperonins. (**A**) The atomic structure of a GroEL monomer (pdb 1KP8), with the equatorial domain (green), apical domain (purple), and intermediate domain (blue). (**B**,**C**) Two orthogonal views of the atomic structure of a Type I chaperonin oligomer in its open (**B**) and closed (**C**) conformations, in the latter case induced by the presence of the small oligomer GroES (red and one of the monomers in orange). The atomic structures correspond to GroEL (pdb 1KP8) and the GroEL/GroES complex (pdb 1AON), respectively. The dashed lines (red) mark the surface formed by the apical domains that is responsible for interaction with misfolded proteins. (**D**) The atomic structure of a Type II monomer, the thermosome from *Methanococcus maripaludis* (pdb 3IZH). The three domains are coloured as in (**A**). The black arrow indicates the helical extension responsible for closure of the cavity see (**F**). (**E**,**F**) Two orthogonal views of the atomic structure of a Type II chaperonin oligomer (**E**) in its open conformation (thermosome from *Methanococcus maripaludis*; pdb 3IZH) and (**F**) in its closed conformation (thermosome from Thermococcus strain KS1; pdb 1Q3R). The dashed circles (red) mark the surface formed by the apical domains that is responsible for part of the interactions with misfolded proteins. Bar = 100 Å for B, C, E and F.

Chaperonins are found in all kingdoms of life, and also in viruses [6,7]. Structurally, the majority of chaperonins are cylinders composed of two back-to-back rings, each of which is an oligomer of ~60 kDa proteins (Figure 1). Each monomer has three domains (Figure 1A,D): (1) the equatorial domain, which hosts the ATP-binding site and is responsible for all inter-ring connections and most of the intra-ring interactions; (2) the apical domain, at the entrance of the ring cavity, which hosts the recognition sites for client proteins; and (3) the intermediate domain, which transduces signals from the equatorial domain (resulting from ATP binding and hydrolysis) to affect the large conformational changes in the apical domain corresponding to the open and closed forms.

Historically, chaperonins have been classified into two types, those of bacterial and endosymbiotic origin (Type I) and those found in archaea and in the eukaryotic cytosol (Type II). Type I are usually homo-heptameric rings, and the best-studied member is the chaperonin GroEL from *Escherichia coli* (Figure 1A–C) [8]. Type II chaperonins, in particular those of archaeal origin (usually named thermosomes), are mostly octameric rings made up of one or two different subunits. Occasionally, nonameric rings have been found, usually composed of three different subunits. The most complex of the Type II chaperonins is the eukaryotic cytosolic chaperonin, termed CCT or TRiC, which contains eight different subunits (Figure 1D–F) [9,10,11]. Although the two chaperonin types are structurally similar, they have two major differences which carry important consequences. Firstly, there are differences in how the subunits of opposite rings interact, with Type I showing a 1:2 subunit arrangement, whereas Type II has a 1:1 arrangement (compare Figure 1B,D). This has a clear consequence on intra- and inter-ring signalling, with clear positive and negative cooperativity among the subunits of a ring and between rings, respectively, in Type I chaperonins, whereas intra- and inter-ring cooperativities have not been unequivocally demonstrated in Type II chaperonins [7,12].

The second important difference between the two chaperonin types involves the manner in which the closed conformation is induced. In Type I, the closed conformation is achieved after ATP binding by capping of the ring with a small heptamer (termed cochaperonin or Hsp10 [GroES in *E. coli*]), which forces the release of misfolded protein, which was previously bound to the apical domains, into the chaperonin cavity (Figure 1C) [13]. In Type II chaperonins, closure of the cavity is executed by a small helical extension of the apical domain (see arrow in Figure 1D), which seals the cavity after ATP binding and hydrolysis in the equatorial domain (Figure 1F) [14].

The conspicuous properties of this structure (a cylinder containing a cavity that allows confinement) and the potential to control its closure and aperture have inspired a number of nanotechnological applications that will be described below.

## 2. Chaperonins as Nanotechnological Devices

The peculiar structure of chaperonins, with a cavity entrance of up to ~80 Å and volume large enough (70–130,000 Å^3^, depending on the chaperonin) to accommodate chemical reagents or proteins up to ~100 kDa, together with the possibility of closing and opening their entrance through an ATP-controlled mechanism (Figure 1), has been cleverly exploited for different purposes.

### 2.1. Chaperonins as Nanocarriers

**Figure 2:**
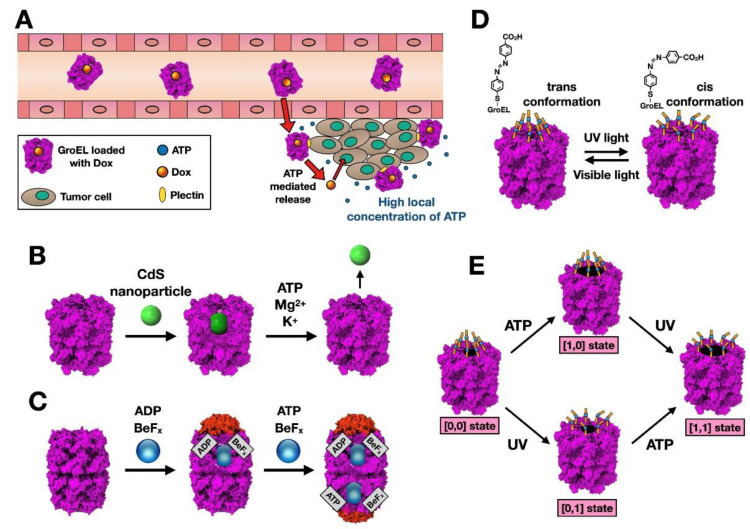
Nanotechnological applications for Type I chaperonins. (**A**) Proposed mechanism of action for GroEL-Dox in drug delivery and release to tumor cells. Modified from [15]. (**B**) The chaperonin-enclosed CdS nanoparticles and their ATP-induced release. As shown, CdS nanoparticles can be encapsulated inside of the chaperonin, and released in the presence of ATP and different ions. Modified from [16]. (**C**) The reaction scheme of nanoparticles encapsulation in the GroEL/GroES complex. In the presence of ADP and BeFx, a 1:1 GroEL:GroES (“bullet”) is formed, while incubation with ATP and BeFx induces the formation of a 1:2 GroEL:GroES complex (“football”). This allows the possibility of caging different nanoparticles in each of the chaperonin’s cavities. Modified from [17]. (**D**) Structural changes induced by the *trans-cis* isomerization of azobenzene-modified GroEL. (**E**) ‘AND’ logic response of azobenzene-modified GroEL to ATP (which induces the chaperonin to an open conformation) and UV (which induces opening of the chemical gate). The chaperonin cavity opens efficiently only in the presence of both stimuli; modified from [19].

In the case of Type I chaperonins, a non-modified GroEL has been tested as a nanocarrier by loading GroEL with Doxorubicin (Dox), a hydrophobic antitumor drug [15] (Figure 2A). In this work, the authors observed how GroEL was able to protect Dox from unwanted degradation in the blood until the chaperonin reached the tumour, where GroEL interacts with plectin, which is highly expressed on the membranes of the tumour cells. Dox release was subsequently induced by the high ATP concentration of this environment (Figure 2A). Yuan et al. took advantage of the high affinity of GroEL for plectin, but the chaperonin usually must be modified for use in more general purposes. GroEL and another Type I chaperonin (that of the eubacteria *Thermus thermophilus*) have been used to carry cadmium sulfide (CdS) nanoparticles (20–40 Å) [16]. These chaperonin-enclosed nanoparticles are well electronically isolated and can retain their properties for more than a year, being released after ATP binding and hydrolysis (Figure 2B). Another way to stably isolate cargo in the chaperonin cavity is by capping it with the cochaperonin GroES. Yoda and Koike-Takeshita [17] have cleverly used a GroEL double mutant with modifications in its equatorial region (Figure 1A), which hydrolyses ATP at a much slower rate and therefore maintains GroES bound to GroEL (Figure 2C). Incubation of the chaperonin first with nanoparticles made of platinum (PtNPs), Au (AuNPs), or a mixture of Fe and Pt (FePtNPs) has successfully capped nanoparticles in both rings at the same time, generating what has been termed as symmetric GroEL:GroES complexes (more commonly known as “footballs”) (Figure 2C). The authors went even further by using GroEL in a more elaborate approach. It has been previously shown that the use of fluoroberylate (BeFx) can mimic binding of the phosphate part of the GroEL-bound nucleotide [18]. In the presence of ATP, BeFx stops the functional turnover of GroEL by preventing GroES release and produces a symmetrical complex in which both GroEL rings contain ATP-BeFx (a “football”). With ADP, BeFx can bind GroES to form a stable 1:1 GroEL:GroES complex (known as a “bullet”). Yoda and Koike-Takeshita [17] have made use of this differential behaviour of BeFx with both nucleotides, first generating a “bullet” with ADP and BeFx with a certain nanoparticle encapsulated, and then enclosing a different nanoparticle in the other cavity by incubating it with ATP and BeFx (Figure 2C).

In some cases, ATP control of the chaperonin may not be desired due to its ubiquity in the cell, and a more controllable approach is required. This is the case in which GroEL was transformed to incorporate an “AND” logic gate [19]. This was done by first removing all the Cys residues of the chaperonin, and then introducing one Cys in the apical domain of GroEL, at the entrance of the cavity (Figure 2D). Subsequently, the SH group was functionalized with an azobenzene derivative (which undergoes *trans-cis* isomerization when exposed to UV radiation), therefore inducing a change in the shape and polarity at the entrance of the chaperonin cavity. Use of this artificial chemical switch in conjunction with the natural one (ATP) resulted in four different conformations (Figure 2E). The authors found that, while the modified chaperonin is able to recognize and trap misfolded proteins, the presence of only one stimulus results in very slow or no release, and thus no refolding of the trapped protein. Only in the presence of both ATP and UV is there an efficient release, the former setting in motion the aperture of the cavity and the latter generating a more polar *cis*-conformation that allows proper exit of the enclosed protein.

**Figure 3:**
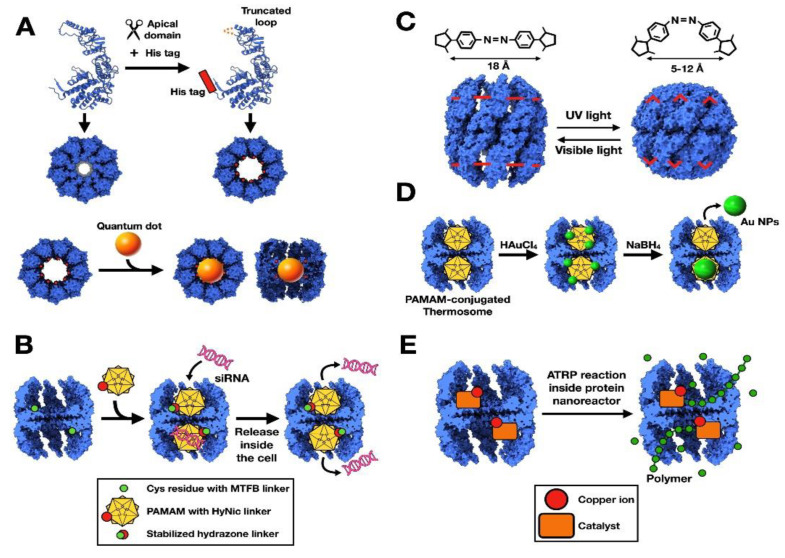
Nanotechnological applications for Type II chaperonins. (**A**) Strategy for the preparation of a complex between a modified chaperonin and a quantum dot [22]. The cavity entrance is widened by removal of the helical protrusion, and a His tag is placed in its interior, at one of chaperonin sequence ends. (**B**) Strategy followed by Nussbaumer et al. [20] to modify a Type II chaperonin (the β-only *Thermoplasma acidophilum* thermosome) so that it could be used as a siRNA carrier. (**C**) Structural changes induced in the thermosome from *Methanococcus maripaludis* by isomerization of the azobenzene dimaleimide (top), which reacts with two Cys residues in opposite positions of the equatorial domains of two adjacent subunits, which in response to light leads to closure or aperture of the cavity (bottom); modified from [23]. (**D**) Section of the thermosome structure from *Thermoplasma acidophilum* in the open conformation, in which PAMAM has been conjugated to Cys residues introduced in the chaperonin cavity. The scheme shows how AuNPs are formed using dendritic polymers as nucleation sites [24]. (**E**) The chaperonin cavity (shown in section) can be used as a nanoreactor for atom-transfer radical polymerization, as described in [25].

Type II chaperonins have also been tested as nanocarriers for biomedical purposes [20]. An interesting experiment was carried out with the thermosome from *Sulfolobus shibatae*, whose rings are nonamers made up of three copies of alternating α, β, and γ subunits [21]. The idea for this archaeal chaperonin was to use it as a quantum dot (QD) nanocarrier [22]. For this, the authors generated a whole oligomer using only the β subunit and then carried out two modifications (Figure 3A). First, they removed a 27-residue loop of the apical domain that is located at the entrance of the cavity to make it wider, and then they introduced a His-tag in the N-terminus, which is physically located in the interior of the cavity. With these modifications, soluble and fluorescent QDs made of zinc sulfide-coated cadmium selenide (CdSe-ZnS) could be trapped and confined in the thermosome cavity (Figure 3A), acquiring photoluminiscent properties. The authors went further by introducing a Cys residue at the tip of the apical domain, which they used to bind a biotin molecule. This biotin-modified QD-enclosed thermosome can be recovered with streptavidin-coated magnetic beads and therefore be used as a sensor for specific targets [22].

Another example of Type II chaperonins modified for biotechnological purposes is the thermosome of *Thermoplasma acidophilum*, composed of two homologous subunits, α and β, which alternate in an octameric ring. In this case, the ring can also be formed by β subunit homo-oligomerization, such that all the Cys residues of this subunit are removed from the sequence and a specific one is inserted within the thermosome cavity [20] (Figure 3B). These residues were made to react with the heterobifunctional linker maleimido trioxa-6-formylbenzamide (MTFB). Additionally, the polycationic dendrimer poly(amidoamine) (PAMAM) could be linked to the modified chaperonin, with the dendrimers enclosed in the cavity used to complex siRNA through electrostatic interactions. The siRNA is protected within the chaperonin cavity from RNase attack, and could be delivered to U87 cells inducing gene silencing. The process was made even more efficient by adding the cell-penetrating peptide TAT (QPPRRRQRRKKRG) to the modified chaperonin, which aided in cell uptake and subsequent siRNA delivery [26]. 

Type II chaperonins have an additional advantage over Type I for their use as nanocontainers: the fact that complete closure of the cavity does not require a cochaperonin (Figure 1C), but rather is possible thanks to the great conformational changes that place a helical extension at the entrance of the cavity, occluding it (Figure 1F). A very elegant idea by Hoersch et al. [23] was to place a photoactivable switch at a site where the movement induced by the isomerization process prompted large rearrangements of the chaperonin monomers that lead to the aperture or closure of the cavity. The authors removed all the Cys residues from the *Methanococcus maripaludis* chaperonin and introduced two new ones in its equatorial domain, which subsequently reacted with a photoswitchable azobenzene molecule. Then, the light-triggered *cis-trans* isomerization induced the closure or aperture of the chaperonin cavity (Figure 3C). These approaches constitute a promising start for generation of nanocarriers that can deliver a pharmacological molecule in a controlled manner. However, when dealing in biomedical applications, the use of the above-mentioned chaperonins presents limitations due to issues with immunocompatibility. To avoid this, the human version of the eukaryotic cytosolic chaperonin CCT seems to be an ideal candidate; however, this chaperonin is quite complex, as each of the two rings is constituted by eight different, albeit homologous subunits (CCT1-8) that are arranged in a fixed position [7,27]. CCT seems to act on a large set of substrates (though it is not as promiscuous as Type I chaperonins or the thermosomes), and its functional cycle and mechanism of action upon the different substrates are not fully understood. All this (together with the difficulty of artificial generation of oligomers composed of eight different proteins) makes CCT a difficult chaperonin to apply to technological challenges. However, it has been shown that two of the subunits (CCT4 and CCT5) are able to self-oligomerize [28], which speaks to the potential use of an artificial human chaperonin as a nanocarrier. 

### 2.2. Chaperonins as Nanoreactors

The chaperonin cage can have other functions, and chaperonins have also been used to construct nanoreactors. Their advantage over much larger standard reactors lies in their small size, which increases reaction speeds by bringing substrates and catalysts closer [29], and above all because the reactions can occur in environments in which they would not normally take place, such as biological systems [30]. Nussbaumer et al. [24] applied a *Thermoplasma acidophilum* chaperonin (with Cys residues introduced in the cavity) as a nanoreactor. Using a similar chemistry to that described above [20], the authors conjugated the dendritic polymer PAMAM to the thermosome. This acted as a nucleation site for tetrachloroaurate anions (HAuCl_4_), which under reductive conditions induces the formation of gold nanoparticles inside the chaperonin cage (Figure 3D). Another interesting use of this chaperonin by the same group was its modification to function as a nanoreactor for atom-transfer radical polymerization (ATRP), a method for controlled reversible-deactivation radical polymerization in which deactivation of the radicals involves reversible atom transfer or reversible group transfer, catalysed by transition-metal complexes [25]. To generate the nanoreactor (Figure 3E), the authors used the Cys residues introduced in the cavity. By performing successive reactions of the modified thermosome with 3-maleimido-6-hydraziniumpyridine hydrochloride (MHPH) and then with N,N,N’,N’-tetraethyldiethylene triamine (TEDETA), a stable bisaryl hydrazone bond was established between the chaperonin and the ligand, which was used to complex Cu^2+^ ions to the modified chaperonin. This chaperonin-bound catalyst was able to positively influence production of narrowly dispersed polymers of N-isopropyl acrylamide and poly(ethylene glycol) methyl ether acrylate. Chaperonin-based nanoreactors like the one described here could control polymerization in a very special way, by directing selectivity for certain monomers or by modulating entry to the folding cavity (e.g., by setting the degree of aperture of the chaperonin entrance; Figure 2D).

### 2.3. Chaperonins as Nanosensors

**Figure 4:**
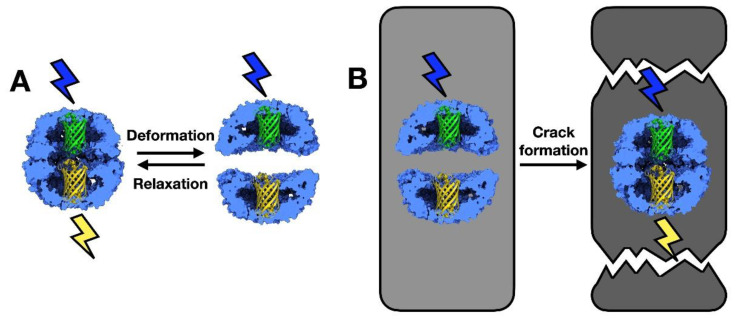
Type II chaperonins used as nanosensors. (**A**) Deformation of a fluorescence-modified chaperonin increases the distance between the donor–acceptor pair, which reduces the FRET signal. (**B**) A fluorescence-modified chaperonin is embedded in a polymer and undergoes polymerization and drying stress. Crack formation induces relaxation in the polymer and chaperonin, which can be detected by changes in the emission intensity of the FRET acceptor complex. The two images are modified from [31].

Another interesting technological application for the chaperonins of *Thermoplasma acidophilum* is that of a nanosensor (Figure 4). Bruns et al. [31] used a Cys residue introduced to the interior of the cavity to link proteins of the green fluorescent protein (GFP) family to the chaperonin. This was done by modifying in parallel the Cys residue via the heterobifunctional linker maleimido trioxa-6-formyl benzamide (MTFB) and the GFP by activating a Lys residue with the heterobifunctional linker succinimidyl 6-hydrazinonicotinate acetone hydrazine (SANH). The sensing system relied on the presence of a donor–acceptor pair of fluorescent proteins, each placed in one of the two chaperonin cavities, allowing for Förster resonance energy transfer (FRET) (Figure 4A). The authors used enhanced cyan fluorescent protein (eCFP) as a donor and enhanced yellow fluorescent protein (eYFP) as the receptor [32]. The idea behind this experiment is that compression or relaxation of the material in which the modified chaperonin is inserted should cause a structural deformation and therefore, changes in the FRET signal. To test it, the authors embedded the modified thermosome in a gel generated with acrylamide and the cross-linker N,N’-methylene-bis-acrylamide, like that used in electrophoresis experiments. The gel formed was left to dry and exposed to uniaxial strain until it cracked. Images of the microcracks that formed in the material revealed an increase in FRET signal, which the authors related to relaxation of the stress imposed during polymerization and subsequent drying (Figure 4B). These experiments pave the way for the generation of polymer-protein hybrid materials with fluorescent properties for use as nanosensors [31].

## 3. Chaperonins as High-Order Nanotechnological Devices

The cylindrical, barrel-like shape of the chaperonins (Figure 1) confers these oligomeric structures interesting properties that can be used for nanotechnological purposes, namely the ability to generate two-dimensional ordered arrays or to grow into filaments (nanotubes) of stacked chaperonins [33,34].

**Figure 5:**
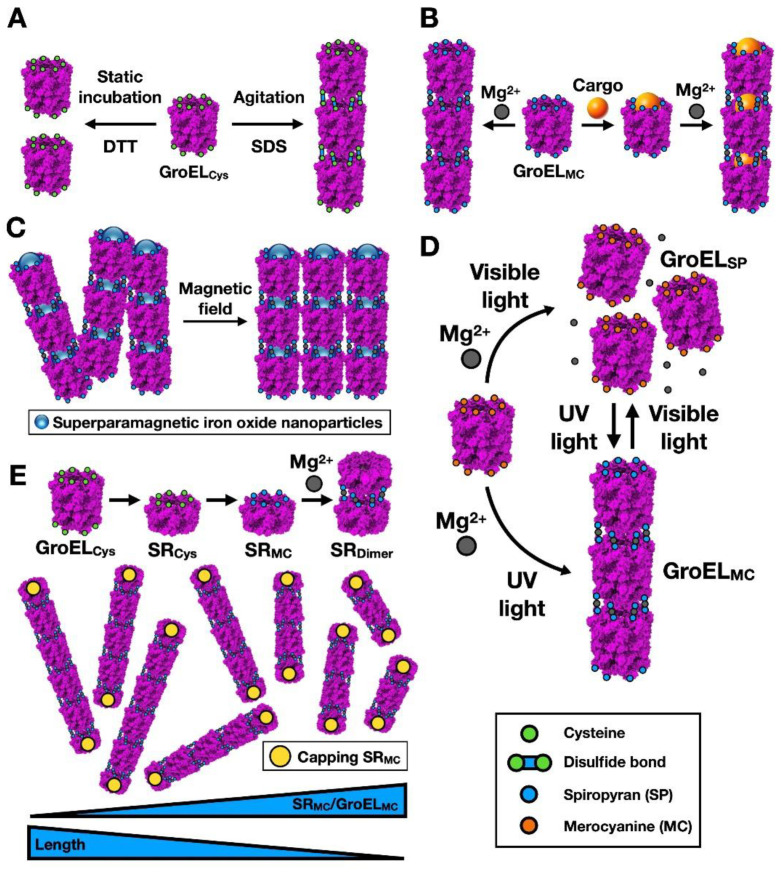
High-order Type I chaperonins for nanotechnological purposes. (**A**) Scheme of the formation of nanofibers using modified GroEL, as described in [35]. A Cys residue (green ball) is placed within the apical domain of the chaperonin, and filaments are formed via disulfide bonds. Filament formation and disassembly is controlled by the redox conditions of the medium. (**B**) Scheme showing the generation of tubular containers based on chemically treated GroEL mutants, according to [36]. A Cys residue (green) was inserted into the apical domains of the chaperonin GroEL, which allows binding of 1′-(maleimidoethyl)spirobenzopyran. Mg^2+^ induces oligomerization of the GroEL cylinders, which can store the cargo (orange sphere) in the locked cavity. (**C**) Illustration of the magneto-induced, lateral assembly of GroEL filaments containing superparamagnetic particles [37]. (**D**) Cartoon depicting the light-induced assembly/disassembly of nanotubes, as described in [39]. (**E**) Top, scheme of the synthesis of the single-ring, merocyanine-modified GroEL version (SR_MC_) and the Mg^2+^-mediated dimer formation. Bottom, filament formation of the MC-modified, two-ring GroEL version, whose length can be limited by the presence of the SR_MC_ version [40].

In the case of Type I chaperonins, one strategy is to promote filament formation by introducing Cys residues within the apical domain, at the entrance of the chaperonin cavity. In a simpler version, two Cys residues were introduced into this region of GroEL, which has a hydrophobic nature due to its role in recognising and trapping misfolded proteins with exposed hydrophobic residues, as a result of the unfolding process (Figure 5A). 

The idea was to achieve filamentous assembly by formation of Cys-Cys bridges between neighbouring GroEL oligomers [35]. Filament formation was improved by rendering the solution mildly hydrophobic by the presence of a detergent such as sodium dodecyl sulphate (SDS) (Figure 5A). Filament formation and disassembly was controlled by the redox conditions. A more sophisticated approach was conducted by the group of Aida [36] to generate large, multiple nanocontainers (Figure 5B). The authors also modified GroEL by introducing in this case a single Cys residue into the apical domain of each of the monomers, at the entrance of the chaperonin cavity. They subsequently reacted those Cys residues with a merocyanin maleimide (MC), which in the presence of Mg^2+^ induced GroEL filaments. The strategy was to cage the cargo [36,37] before filament formation, which was triggered by the presence of Mg^2+^. The presence of ATP (and its hydrolysis) induces conformational changes in the chaperonin, which in turn generates a mechanical force and subsequent disassembly of the chaperonin filaments. The rate of disassembly is dependent on ATP concentration, which points to the possibility of selective cargo release, in particular in tumour tissues. This strategy has been used for intracellular distribution of a cargo drug by boronic acid-functionalized chaperonin filaments that are able to penetrate cells [38].

The same authors have gone even further, using superparamagnetic nanoparticles (SNPs) made of iron oxide as a cargo, which can be chemically modified to become coated with different compounds such as fluorescent labels (Figure 5C). In the presence of a moderate magnetic field (0.5 T), the SNP-containing filaments can be attracted and interact laterally to form large bundles [37].

A more sophisticated strategy has been devised by the same authors to control nanotube assembly and disassembly that is independent of cellular conditions (such as ATP concentration). Using the same GroEL modified with a Cys residue at the entrance of the chaperonin cavity, they succeeded in creating a photoreconfigurable filament by linking spiropyran to the Cys residues (GroEL-SP) (Figure 5D) [39]. This non-ionic compound is able to reversibly isomerize upon UV treatment into the ionic merocyanine (GroEL-MC), which can in turn induce GroEL-MC nanotubes in the presence of Mg^2+^. These nanotubes can disassemble into monomers by visible light treatment, which reconverts GroEL-MC into GroEL-SP (Figure 5D). Despite its own problems related to its use in vivo, light has the advantage of having a non-invasive nature and can provide a very specific treatment, both in time and space. Finally, a mutant of the GroEL-MC oligomer that forms a single ring (SR-MC; by mutation of the residues involved in the GroEL ring-ring interaction; [41]) was used to cap and block filament formation, controlling nanotube length by use of a proper ratio of GroEL-MC and SR-MC mutants (Figure 5E) [40]. The authors found that smaller filaments were much more efficient in cellular intake than the longer ones, which is a possible pharmacological advantage.

**Figure 6:**
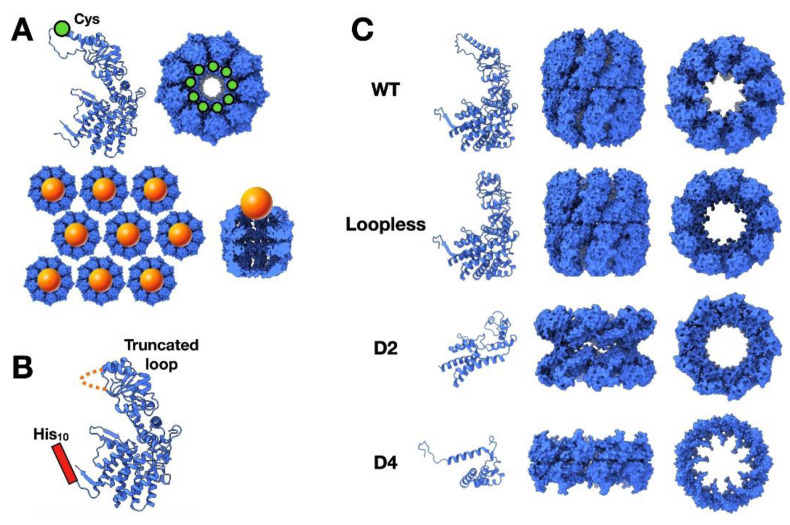
High-order Type II chaperonins for nanotechnological purposes. (**A**) Top, scheme of the mutation carried out in the *Sulfolobus shibatae* thermosome, in which a Cys residue is introduced at the tip of the helical protrusion. Bottom, conditions found for the generation of two-dimensional, ordered arrays [47]. (**B**) A version of the prior scheme, in which the same authors replaced the helical protrusion with a His tag in the interior of the cavity, thus allowing a wider access [48]. (**C**) Representations of some of the different mutants generated by [49]. Left, atomic model of the different variants. Centre and right, two orthogonal views of the oligomer formed with the different variants.

As happens with other biomolecules of regular structure such as DNA and icosahedral viruses, Type II chaperonins have been used as templates for the patterning of arrays [42,43]. These chaperonins have an added advantage over those of Type I in that they are prone to form ordered two-dimensional arrays [44,45,46]. Using this property, the nonameric thermosome from *Sulfolobus shibatae* was modified to introduce a Cys residue (as in many of the cases described above), this time within the typical helical extension of Type II chaperonins (Figure 6A, left). A thiol group was added through a Cys residue, such that it could interact after generation of the two-dimensional array with QDs made of CdSe-ZnS nanoparticles (Figure 6A, top), thus generating a well-ordered array of nanoparticles located at the entrance of the thermosome cavity (Figure 6A, bottom) [47]. An improvement on this protocol allowed this type of array to be used as a technique for patterning nanoparticles inside the chaperonin cavity [48]. In this case, the helical extension of the thermosome was removed to widen the entrance of the chaperonin cavity (Figure 6B). Then a His_10_ tag was inserted at the N-terminus of the sequence, which is located in the chaperonin cavity. This smaller chamber has high affinity for metal ions, due to the abundance of His residues (at least 180). When the chaperonin arrays are incubated with Pd^2+^, the chaperonin cavities become sites for the initiation of chemical reduction of magnetic transition metals such as Ni^2+^ or Co^2+^ (from precursor salts). The reaction gives rise to Ni-Pd or Co-Pd nanoparticles, with sizes limited by that of the chaperonin cavity. These artificially, chaperonin-driven arrays could be potentially used for other nanotechnological purposes such as the controlled growth of other materials [48], and may be modified to further widen the cavity entrance by removing part of the apical and intermediate domains, without loss of the general nonameric, double-ring structure (Figure 6C) [49].

This review intends to give an account of the nanotechnological applications that have been devised for the chaperonins over the last 20 years, both in the field of biology and material science in the investigation of new biomaterials that could act as sensors, reactors, or nanocarriers of materials with biotechnological or pharmacological or functions, in particular in the field of cancer therapy.
